# Our Recent Dead

**Published:** 1893-05

**Authors:** 


### OUR RECENT DEAD.

THE dental profession in the past two years has had to mourn the loss of many of its most active workers. The ever-present reaper has spared neither the aged veteran, crowned with honor, nor the earnest youth, ambitious to mark a path through the mazes of a world's civilization.

The recent loss of two such men as Watt and Allport recalls the period in which they were recognized leaders, an era replete with developments from crude surroundings to a professional life worthy of the progressive age of which we form a part. Their work was well done, and their eyes were able before the close to look forward and view the promised land which they had struggled through so many years to reach,—the condition of a self-sustaining and consistent professional life. They have passed away, and the dental profession honors their memory. It will be given to few to acquire the same dominating power over men maintained for years by these two prominent workers. The biographer will find it difficult, perhaps, to analyze this influence; but it existed, and now, as we write, it seems as though the loss of such leadership inspires a sense of loneliness. The mantles of the Fathers are falling one by one. Who is there to pick them up and wear them with equal power and honor?

We may stand by the graves of our honored dead and mourn the loss of their wisdom, but when we are called to pay the melancholy tribute to the young and active spirits who have faded from life and professional work, our hearts sink at the seeming sacrifice.

When we recall the untiring labor of Frederick A. Levy, we are reminded that, though "leaves have their time to fall," the autumn of this life had not been reached. It is hard to be reconciled to the inevitable, and to feel that in the great struggle still upon us his genial presence will no longer be with us.

Again the funeral-bells are tolling the requiem of a still younger man, one who had just begun to make an impress upon the scientific thought of his colleagues; and if the reaper ever succeeded in securing a "shining mark," he was successful when William F. Rehfuss was removed from our midst.

This loss comes very near to the writer's best life. He was a student among students, and his laborious work, concentrated within the compass of a few years, sapped the life forces, and he has fallen a devotee at the shrine of incessant labor. There is probably no other young man in the dental profession, and but few in any other, who has succeeded in accomplishing as much at his twenty-sixth year. His work on "Dental Jurisprudence" is a marvel of industry, and will carry his name forward on the roll of fame to many who will never know of his personality. It is impossible to feel satisfied with this loss. While we may rejoice in the work accomplished, it is accompanied with the sad reflection that it is to that labor to which we must attribute his destruction. It is the ever suggestive lesson that there is a limit to human endurance.

The bier is ever moving towards the cemetery. The mourners are ever asking the old, old question, "What is life that it should fade, and why are the voices of those we knew hushed forever? Where is the compensation?"

We have no answer to give to such, but to those who appreciate faithful work and honest endeavor the life of Dr. Rehfuss will ever remain an inspiration and incentive to greater perfection.

The carolling of birds will herald the coming spring-time, the flowers will bloom and shed their fragrance over our dead companions, the places which knew them on earth will be vacant forever, yet the impressions of thought which they made will be an ever-enduring presence, illuminating, elevating, and extending the area of work still left us to perform.

Our dead have passed from sight, but not from memory, and as the gate of the cemetery closes and the body passes on, "ashes to ashes," this consolation is left with us, that this life

> "Is but a suburb of the life elysian,
> Whose portal we call death."

